# Fascia Iliaca vs. Combined Iliaca Blocks for Proximal Hip Fractures in the Emergency Department

**DOI:** 10.5811/westjem.48675

**Published:** 2026-02-01

**Authors:** Joseph Betcher, Alex Glogoza, Austin Poulson, Oliver Snyder, Benjamin Black

**Affiliations:** * Michigan State University, Lake Michigan Emergency Specialists, Muskegon, Michigan; † Michigan State University, West Michigan Emergency Medicine Residency, Muskegon, Michigan

## Abstract

**Introduction:**

Over 335,000 adults are hospitalized annually for proximal hip fractures, with the incidence of these injuries increasing as the population ages. Our objective in this study was to compare pain scores of patients with proximal hip fracture 30 minutes after undergoing a combined fascia iliaca plus femoral nerve block vs standard fascia iliaca block.

**Methods:**

We performed a retrospective cohort study including all isolated proximal hip fracture patients ≥ 18 years of age who underwent regional anesthesia by ultrasound fellowship-trained emergency physicians in a community hospital emergency department between January 1, 2022–September 26, 2024. We excluded patients with distal femur fractures, those who had received additional pain medications within 30 minutes of the block, or those who could not reliably relay a pain score. The primary outcome was subjective pain scores (scale 1–10) after undergoing regional anesthesia.

**Results:**

Of 89 patients who underwent regional anesthesia for proximal hip fracture, 20 were excluded. A total of 31 fascia iliaca blocks and 38 combined blocks were performed. Patient age, weight, and pre-procedure pain scores were similar between the groups. Females were more predominant in the fascia iliaca block group (67.7% vs 42.1%; *P* = .03). On average, patients who received the combined block rated their post-procedure pain score 1.4 points lower than those who received a fascia block (3.8 vs 5.2/10, *P* = .01). This finding was consistent when controlling for sex and pre-procedure pain scores (β: 1.5; 95% CI, 0.6–2.4).

**Conclusion:**

Undergoing combined fascia iliaca plus femoral nerve block was associated with lower pain scores after 30 minutes compared to isolated fascia iliaca block in patients with proximal hip fractures. These patients may benefit from using this single-injection procedure for improved pain control.

## INTRODUCTION

Over 335,000 adults are hospitalized annually for proximal hip fractures, with the incidence of these injuries increasing as the population ages.^1^,^2^ Unfortunately, the elderly population that is at increased risk for suffering these injuries is also at increased risk for delirium, decreased postoperative mobility, and lower health-related quality of life.^3^,^4^ Regional anesthesia has demonstrated efficacy in this demographic by providing improved pain control, reducing systemic analgesia requirements, and decreasing the incidence of delirium.^4^,^5^ Based on available evidence, clinical practice guidelines from the American Academy of Orthopaedic Surgeons gives a strong recommendation for use of regional anesthesia in patients with proximal hip fractures.^6^–^8^

Fascia iliaca blocks, femoral nerve blocks, and pericapsular nerve group (PENG) blocks can be used as regional anesthesia techniques to provide pain relief to these injuries.^9^,^10^ Multiple studies have evaluated pain control with PENG blocks vs. fascia iliaca blocks.^11^–^13^ Perioperative pain control with fascia iliaca block vs. femoral nerve block has been shown to be equivalent when studied in total hip and knee arthroplasties.^14^ The two types of nerve blocks are located in a closely related anatomical region, making it feasible for the physician to perform a combined block using a single needle entry within the affected hip.

**Image 1:**
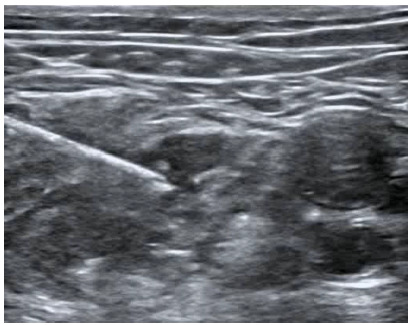
Fascia iliaca block, deposition of anesthetic under fascial plane.

**Image 2:**
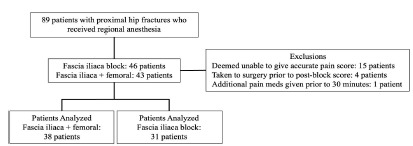
Femoral nerve block, deposition of anesthetic lateral to femoral nerve.

The fascia iliaca block is administered as a plane block, involving the injection of a high volume of diluted anesthetic beneath the fascia iliaca (Image 1). Given its proximity to the femoral nerve, we theorized that after injecting beneath the fascial sheath, the needle could be advanced directly toward the femoral nerve to perform an additional targeted femoral nerve block (Image 2). This may confirm the benefit of both blocks in a single injection, offering increased pain control for patients. Furthermore, both blocks have been proven safe for patients on anticoagulants, making them a suitable option for the elderly who take these medications.^15^

There is significant evidence that regional anesthesia can improve pain scores, patient outcomes, and decrease narcotic use for patients who present with proximal hip fractures.^4^,^16^,^17^ Regional anesthesia in proximal femur fractures also decreases fracture-related morbidity, such as delirium and chest infections.^16^ There is limited knowledge about the effectiveness of performing both blocks together and whether this approach provides greater pain control than an isolated fascia iliaca block in patients with a proximal femur fracture.

In this study we aimed to assess whether a combined fascia iliaca and femoral nerve block is associated with increased postoperative pain control compared to the standard fascia iliaca block alone.

**Population Health Research Capsule**

What do we already know about this issue?

*Regional anesthesia for patients has been shown to lead to increased pain control and to decrease overall complications*.

What was the research question?

*Will a fascia iliaca block combined with a femoral nerve block lead to increased pain control?*

What was the major finding of the study?

*Patients with the combined block had a pain score 1.4 points lower than those who underwent standard fascia iliaca block (3.8 vs 5.2/10, P = .01)*.

How does this improve population health?

*Use of the combined fascia and femoral block may lead to overall pain control for patients with proximal hip fractures*.

## METHODS

This retrospective cohort study reviewed all patients who received regional anesthesia after sustaining a proximal hip fracture between January 1, 2022–September 26, 2024. The institutional review board approved the research project before any data were collected. We performed this study, which followed the STROBE guidelines for retrospective cohort studies to enhance the transparency and rigor of reporting, at a community-based emergency department (ED) designation Level II trauma center.

**Figure 1:**
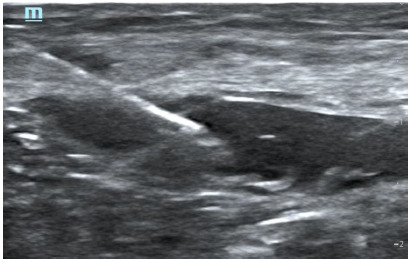
Patient-inclusion flow diagram of a study designed to assess whether a combined fascia iliaca and femoral nerve block is associated with increased post-procedural pain control compared to the standard fascia iliaca block alone.

Inclusion criteria required that patients be at least 18 years of age at the time of presentation, have sustained a proximal hip fracture, specifically femoral neck and intertrochanteric fractures, and have undergone regional anesthesia performed by an emergency physician within the ED. Exclusion criteria included isolated pelvic fractures, midshaft or distal femur fractures, or cases in which the anesthesia department administered regional anesthesia (Figure 1). We excluded from the analysis patients who were deemed unreliable to give an accurate pain score by the physician administering the block, those who were brought to the operating room for surgical fixation in < 30 minutes post-block, or who had received additional medication for pain control in < 30 minutes post-block.

Patients in this study received regional anesthesia using one of two block techniques: isolated fascia iliaca nerve block; or combined fascia iliaca with femoral nerve block. Patients receiving a fascia iliaca block received 20 cc 0.5% ropivacaine combined with 20 cc sterile saline. Patients who underwent a combined block received 25 cc 0.5% ropivacaine combined with 25 cc sterile saline. The combined block was performed through a single injection site; 30 cc of the solution was first administered for the fascia iliaca block, after which the needle was advanced toward the femoral nerve, and the remaining 20 cc were deposited within 1 cm of the nerve. The volumes of anesthetic were an agreed-upon protocol between the anesthesia and emergency departments prior to initiation of this study. One ultrasound fellowship-trained emergency physician directly supervised and assisted all procedures to ensure consistency in block technique. All blocks were performed under the real-time, in-plane guidance of a Mindray TE^7^ ultrasound machine (Shenzhen Mindray Bio-Medical Electronics Co, Ltd, Shenzhen, China).

We collected data via retrospective chart review using our ED’s electronic health record; data included pre- and post-procedure pain scores, patient age, weight, sex, and which type of block was performed. To perform an optimal chart review, previous literature from Worster and Bledsoe^18^ was referenced: We used the principles of abstractor training; case selection criteria; variable definition; performance monitoring; medical record identification; sampling methods; missing-data management plan; and institutional review board approval. Our primary measured outcome was patient-rated pain scores (1–10) 30 minutes after performing regional anesthesia.

We calculated summary statistics, and we used the *t*-test to compare quantitative data, which are expressed as the mean+standard deviation. Results from a linear regression are reported as beta coefficient (β) and 95% confidence interval. Nominal data were analyzed using the chi-square or Fisher exact test and are expressed as a percentage. Significance was assessed at *P* < .05.

## RESULTS

The study sample included 38 patients who received a combined fascia iliaca and femoral nerve block, while 31 patients received a fascia iliaca block alone. Baseline demographics and preoperative pain scores were mostly similar between the two study groups (Table 1). However, there was a significantly higher proportion of female patients in the fascia iliaca-only group (67.7%) compared to the combined fascia iliaca and femoral nerve block group (42.1%).

Average pain scores decreased by four points in the combined fascia iliaca and femoral nerve block and by 2.6 points in the fascia iliaca block-only group. In the primary analysis, patients who received the combined fascia iliaca and femoral nerve block reported significantly lower post-procedure pain scores than those who received the fascia iliaca block alone, mean difference 1.4 points (95% CI, 0.3–2.5) (Table 1). These findings remained consistent when adjusting for sex and pre-procedure pain scores (β: 1.5; 95% CI, 0.6–2.4).

## DISCUSSION

Pain control in the ED setting remains a top priority for patients who present with proximal hip fractures. The fascia iliaca nerve block has been proven to be effective at treating fracture pain and has been a mainstay of treatment for decades, but it is often insufficient at providing adequate pain relief in isolation, requiring adjunct treatment with other pain control modalities.^10^,^14^ This study demonstrates that a traditional fascia iliaca combined with a femoral nerve block was associated with increased pain control compared to an isolated fascia iliaca block in patients with proximal hip fractures, with an average reported difference of 1.4 points on a subjective (1–10) pain scale.

Nuthep et al had previously trialed a combination of PENG + suprainguinal fascia iliaca block but found no improved pain control with the addition of the PENG block.^19^ The theory behind blocking the femoral nerve at two separate locations is that it may enhance the analgesic effect, although this was not previously demonstrated by Nuthep.^20^ Those authors hypothesized that the lack of increased pain control was potentially due to both block types targeting the femoral nerve, as the PENG targets the articular branches, while the fascia iliaca targets closer to the inguinal ligament. They also noted that their small sample size may have led to decreased statistical power, and the inability to show significant differences. Similarly, Zheng et al compared intra-articular injections postoperatively with the addition of a PENG block and found that it did not improve overall pain control as compared to intra-articular blocks alone.^21^

Seker et al conducted a study comparing PENG block alone with PENG block combined with lateral femoral cutaneous nerve block (LCFN), specifically evaluating postoperative opioid consumption, and reported no significant differences with the addition of the LCFN block. However, their investigation primarily focused on postoperative pain outcomes, whereas in the present study we emphasize preoperative pain scores.^22^ A recent case series by Duan examined the addition of a pericapsular hip block to the PENG block and reported effective analgesia without complications; however, the Duan series was not conducted as a comparative study.^23^

To our knowledge, our study is the first to compare a fascia iliaca vs. a fascia iliaca combination approach with femoral nerve block. While the fascia iliaca block may provide coverage to larger distribution—targeting the femoral nerve, lateral cutaneous nerve, and potentially the obturator nerve—it can be overall expectedly less specific to the femoral nerve.^24^ When using the fascia iliaca block, the anesthetic must traverse a larger fascial compartment and may disperse variably; thus, it may not consistently provide dense or reliable coverage of the femoral nerve.^24^ Consequently, although the fascia iliaca block offers the advantage of addressing multiple nerve territories, its effectiveness in ensuring precise and sustained femoral nerve blockade can be limited compared to more targeted approaches.

We hypothesized this combined technique would help to ensure adequate coverage of the femoral nerve and that, ideally, the enhanced analgesia provided by the combined block technique would result in a further reduction in the need for systemic analgesics, particularly in elderly patients who are at increased risk for complications such as postoperative delirium and impaired mobility. This technique was agreed upon by both the anesthesiology and emergency departments and was performed successfully for several years prior to the data collection.

Simultaneous administration of fascia iliaca and femoral nerve blocks offers the advantage of being performed through a single needle-insertion site. After the local anesthetic is injected beneath the fascia iliaca, the needle may be partially withdrawn and subsequently re-advanced toward the inguinal ligament, where the anesthetic is delivered around the femoral nerve. As with all nerve blocks, the femoral nerve block carries inherit risks, including the potential for direct nerve injury; however, the use of slow, in-plane needle advancement under continuous ultrasound guidance can reduce this risk. This technique not only enhances pain management but also minimizes additional patient discomfort and requires minimal effort from the physician. This method, with the observed improvement in pain control, may provide an additional tool for physicians caring for this patient population.

## LIMITATIONS

Our study has multiple limitations. First, it was a retrospective review. Although baseline characteristics were similar for each of our treatment groups, the retrospective nature of our study was subject to selection bias and did not allow for randomization. However, we did control for potential confounders in our primary analysis and found consistent results in post-procedure pain scores. Future prospective and randomized trials could reduce any possible impact of these biases.

Assessing primary outcomes using pain scores presents a challenge due to their subjective nature and the potential for high variability among patients. Prior studies have demonstrated the challenge of using subjective pain assessment.^25^–^27^ Pain perception is influenced by numerous factors, including individual pain tolerance, psychological state, and even external influences such as environment and medication effects.^27^,^28^ Additionally, differences in communication abilities, particularly in elderly patients or those with cognitive impairments, further diminish the reliability of subjective pain assessments. Our study excluded patients who were unable to provide reliable scores upon prompting. The same physician who performed all blocks determined pain score reliability across all patients to maintain consistency in assessment criteria.

An additional confounding variable pertains to the volume of lidocaine administered. Patients who received the combined fascia iliaca and femoral nerve blocks were given a slightly greater total volume of lidocaine compared to those who underwent fascia iliaca block alone. This dosing strategy was predetermined and established through consensus between the emergency and anesthesia departments. The increased volume of local anesthetic in the combined block group may have contributed to the observed differences in pain control. Future research should standardize the dosage of lidocaine to enable more accurate comparisons between treatment groups.

## CONCLUSION

The use of a combined fascia iliaca and femoral nerve block for proximal hip fractures was associated with improved pain scores, supporting its role as an effective analgesic strategy for the elderly patient population. Additional randomized controlled trials comparing these techniques could provide further insight into their efficacy in pain management.

## Figures and Tables

**Table 1:** Patient demographics and outcome comparisons in a retrospective review of fascia iliaca block compared to fascia iliaca + femoral nerve block for pain control.†

	Fascia + Femoral block n = 38	Fascia block n = 31	*P*-value
Age	76.2±13.8	74.6±11.6	.60
Weight (kg)	71.8±19.6	73.7±21.0	.71
Sex, % female	42.1%	67.7%	.03
Pre-score	7.9±2.4	7.8±2.2	.86
Post-score	3.8±2.4	5.2±2.0	.01

† Quantitative data are shown as mean±SD.

*kg*, kilogram.
